# Evaluation of the Antioxidant and Anti-Cancer Potential of Microwave-Assisted *Opuntia humifusa* (Korean Cheonnyencho) Aqueous Extract

**DOI:** 10.3390/cimb47020088

**Published:** 2025-01-31

**Authors:** Poojitha Yanamala, Jeong-Yun Youn, Prakash Thangavel, Ju-Young Moon, Young-Chul Lee

**Affiliations:** 1 Department of BioNano Technology, Gachon University, 1342 Seongnamdaero, Sujeong-gu, Seongnam-si 13120, Republic of Korea; poojiroyal29@gmail.com (P.Y.); yjymjw25@gachon.ac.kr (J.-Y.Y.); prakashjacob47@gmail.com (P.T.); 2 Graduate School of Business Administration, Kwangwoon University, Nuri Hall, 20, Kwangwoon-ro, Nowon-gu, Seoul 01897, Republic of Korea

**Keywords:** colorectal cancer, Korean Cheonnyencho, microwave-assisted extraction, antioxidant

## Abstract

*O. humifusa* (Korean Cheonnyencho), a prickly pear cactus species, has garnered increased attention owing to its rich phytochemical composition and potential health benefits. In this study, the antioxidant and anti-cancer activities of a microwave-assisted aqueous extract derived from O. *humifusa* were investigated, and its phytochemical content was characterized. High-performance liquid chromatography (HPLC) was used to identify various bioactive compounds, including polyphenols, flavonoids, and other antioxidants known for their potential health-promoting properties. Furthermore, the individual compounds in the flavonoids were separated using the HPLC fractionation technique. The antioxidant potential of the aqueous extract was evaluated using 2,2-diphenyl-1-picrylhydrazyl radical scavenging activity. The results demonstrated the significant antioxidant activity of the extract, as evidenced by its ability to scavenge free radicals and effectively reduce oxidized molecules. The experiments involved treating colon cancer cells with varying concentrations of the extract (25 to 125 mg/mL) over a 24-h period, resulting in a remarkable dose-dependent inhibition of cell growth. Notably, this inhibitory effect was absent in HDFa cells, highlighting the potential selectivity of O. *humifusa* in targeting colon cancer cells.

## 1. Introduction

According to the International Association of Cancer Registries [1], colorectal cancer (CRC) is the third most prevalent malignancy worldwide, contributing significantly to cancer-related mortality, with an annual toll of nearly one million lives worldwide. Most CRC cases are associated with random mutations related to various risk or lifestyle factors. In contrast, 10–30% of cases have a familial lineage, and less than 5% exhibit hereditary manifestations [2,3]. Dietary habits rich in meat, cigarette smoke exposure, alcohol consumption, and chronic inflammation in individuals with inflammatory bowel disease (IBD) collectively serve as pivotal independent risk factors for CRC. Highlighting its grim prevalence, CRC ranks as the third most common cause of cancer and the second most common cause of cancer-related deaths globally [4]. Despite improvements in survival rates, metastatic CRC (mCRC) remains a formidable disease, with a five-year survival rate of approximately 14% [5]. Current therapeutic approaches are limited in efficacy against specific CRC types or carry the risk of severe side effects, prompting the integration of novel treatments targeting mCRC as a beneficial addition to the existing therapeutic paradigm [6]. Recently, the emergence of revolutionary technologies, such as whole-genome and single-cell sequencing, the CRISPR/Cas9 system, and the creation of transgenic mouse models, have advanced our understanding of the mechanisms driving CRC metastasis [7]. In turn, this knowledge has led to the development of targeted therapies and immunotherapies capable of circumventing the undesirable cytotoxicity and resistance issues often associated with systemic chemotherapy. Various reviews have offered a comprehensive overview of the available targeted agents and immunotherapies for CRC. It is crucial to note that guidelines continuously evolve in response to the expanding body of clinical trials, shaping the landscape of recommended therapies [8].

Despite extensive efforts to combat malignant diseases using advanced medical techniques, the escalating prevalence of conditions such as cancer and coronary heart disease remains unchecked [9]. Consequently, there is growing interest in exploring the medicinal and physiological advantages fruits and vegetables offer [10]. Numerous studies have investigated the potential of vegetable and fruit consumption as deterrents against cancer [11,12,13,14,15]. This historical record highlights the medicinal applications of plant-based natural products derived from secondary metabolism in plants [12]. Researchers have used modern technology to investigate the pharmacological properties of natural products such as flavonoids, carotenoids, sterols, alkaloids, terpenoids, and vitamins [10,13]. *O. humifusa*, belonging to the Cactaceae family and commonly referred to as Eastern Prickly Pear, stands out as one of the most cold-resistant cactus species (tolerant down to −24 °C) and finds widespread cultivation globally for its diverse uses as an ornamental plant, a source of fruits and vegetables, a forage crop, and a medicinal plant [14]. Notably, it features in the traditional medicinal practices of American, Indian, Mexican, and Korean histories. Certain varieties of cactus fruit boast higher vitamin C content and total antioxidant activities than more familiar fruits such as apples, grapes, and peaches [14,15]. Given that free radicals (superoxide anion radicals, hydroxyl radicals, and nitric oxide) and other reactive species (peroxides and peroxynitrites) contribute to cellular damage in the human body, the antioxidant activities of these fruits play a crucial role in maintaining health and disease prevention [16]. In addition to their antioxidant properties, various cactus species have been posited to exhibit potential health protective effects, including anti-cancer, anti-tumor, and anti-inflammatory properties, as well as preventive measures against chronic diseases both in vitro and *in vivo*. However, there is not much data available on the health-promoting attributes and physiological-protective mechanisms of cactus bio-actives [17,18,19]. The three most prevalent cancers affecting both men and women worldwide are lung, colon, and breast cancers. The incidences of colon and breast cancers are closely associated with dietary habits [20]. Cumulative evidence supports the notion that consumption of *O. humifusa* and its fruits can mitigate the occurrence of colon cancer; however, the extraction of compounds from *O. humifusa* has consistently posed a significant challenge, primarily because of the abundance of saponins. Saponins can interfere with the isolation and purification of other bioactive substances, such as polyphenols and flavonoids, due to their surfactant-like properties. They tend to form foam and emulsions, which complicate the separation process during extraction. This abundance of saponins requires careful selection of extraction methods, such as microwave-assisted extraction or the use of specific solvents, to minimize their interference and ensure the effective recovery of the desired compounds [14,21].

In this study, we aimed to explore the phytochemical profile and bioactive potential of *Opuntia humifusa*, a species known for its medicinal properties. Utilizing a microwave-assisted extraction method, we successfully isolated a polyphenol- and flavonoid-rich aqueous extract, which was chemically characterized and fractionated using HPLC. Our investigation focused on examining the antioxidant capacity through DPPH radical scavenging assays, which demonstrated its significant free-radical neutralization abilities. In addition, we assessed the anti-cancer potential of the extract against HCT116-CCL-247 colon cancer cells. By linking the chemical composition of *O. humifusa* with its bioactivity, this study highlights the potential of this plant as a source of natural antioxidants and anti-cancer agents, providing a basis for further exploration into its therapeutic applications.

## 2. Materials and Methods

### 2.1. Materials and Reagents

Raw *O. humifusa* leaves were grown for three years and harvested in Uiryeong-gu, Gyeongsangnam-do, South Korea. Methanol and phosphoric acid were purchased from Daejung Chemicals and Metals Co., Ltd. (Siheung-si, Gyeonggi-do, South Korea). HCT 116-CCL-247 was purchased from ATCC, HDFa cell lines were purchased from the Korea Cell Line Bank (KCLB) (Seoul, South Korea) and the 2,2-diphenyl-1-picrylhydrazyl (DPPH) assay reagent was purchased from Sigma Aldrich (St. Louis, MO, USA). McCoy’s 5A (Modified) Medium was purchased from Thermo Fisher Scientific, Inc. (Waltham, MA, USA). Throughout the experiments, a Milli-Q Millipore filter system (Millipore Co., Biller-ica, MA, USA) was used to drain deionized (DI) water at 18.2 MΩ. Fetal bovine serum (FBS), trypsin–EDTA, and penicillin–streptomycin solution was purchased from Gibco Laboratories (ThermoFisher, Toronto, Canada). Cell culture flasks were purchased from NEST Scientific (Waltham, MA, USA). All other reagents were of pharmaceutical grade and purchased from Sigma-Aldrich (St Louis, MO, USA). 2.2. Extraction of *O. Humifusa*.

**Figure 1:**
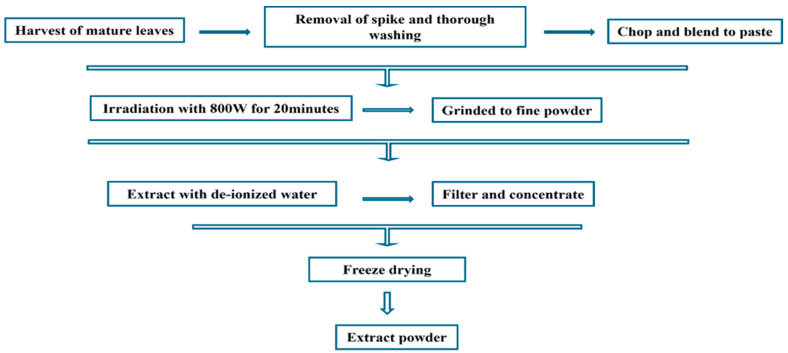
Schematic of the microwave-assisted *O. humifusa* aqueous extract extraction process.

Raw *O. humifusa* leaves were thoroughly washed with water and the thorns removed. The leaves were then air-dried, chopped into small pieces, and blended into a homogeneous paste. The resulting mixture (500 g) was subjected to microwave irradiation at 800 W for 20 min in a standard microwave oven. After irradiation, the mixture was allowed to cool to an ambient temperature, after which it was milled into a powder. This powder was then resuspended in 500 mL of DI water and centrifuged to dissolve the active components and remove insoluble contaminants. To obtain a pure extract, the suspension underwent filtration through a 0.22 μm filter and was concentrated using a rotary evaporator. The final step involved obtaining the microwave-assisted *O. humifusa* aqueous extract (MA-OHE) powder by freeze-drying this solution. The schematic of the *O. humifusa* aqueous extract extraction procedure is shown in Figure 1.

### 2.2. Chemical Characterization of Extracts

A comprehensive and unbiased analysis of flavonoids was conducted using a high-performance liquid chromatography–diode array detection (HPLC-DAD) system, specifically the HPLC Waters Alliance 2695 system. The system comprised a quaternary pump, degasser, automatic sample injector, and column oven. Data processing was facilitated by a Waters 2487 dual spectrophotometric detector using an Empower 2 system. Peak areas were automatically integrated by a computer using the Waters software (version 1.02). Chromatographic separation was executed employing a C18 column (15 cm × 4 mm, 5 µm, Waters), maintaining a column temperature of 30 °C and a flow rate of 1 mL/min. The mobile phase consisted of solvent A (DI water + 0.5% phosphoric acid) and solvent B (MeOH 65% + DI water 34.5 + 0.5% phosphoric acid), which were meticulously prepared and degassed prior to use. This analytical approach ensured a thorough and precise examination of the flavonoids.

### 2.3. Radical Scavenging Activity

To assess the radical scavenging activity, *O. humifusa* aqueous extracts were evaluated using the DPPH assay [22]. Briefly, a 0.1 mM solution of DPPH in methanol was prepared, 0.9 mL of which was added to 0.1 mL of MA-OHE water and raw *O. humifusa* at various concentrations (20, 40, 60, 80, 100, and 120 μg/mL), with ascorbic acid as a standard. The mixtures were vigorously shaken and allowed to stand for 30 min in darkness. This method involves the use of the stable free radical DPPH. When the antioxidant reduces the DPPH, the violet color of the DPPH solution (dissolved in MeOH) changes to yellow; consequently, the alteration in color due to the DPPH radical reaction was monitored by measuring the absorbance at 517 nm, and the antioxidant activity was determined as follows:

(1) $$Scavenging\;Activity\left(\%\right)=\frac{A_{0}-A}{A}*100$$

where A_0_ and A represent the initial and final absorbances, respectively.

### 2.4. Cell Culture

HCT 116-CCL-247, a human colorectal carcinoma cell line, was cultured in McCoy’s 5A (Modified) Medium. HDFa, human dermal fibroblasts (adult), were cultured in Iscove’s Modified Dulbecco’s Medium (IMDM). Both media were supplemented with 10% fetal bovine serum (FBS) and 1% penicillin (100 U/mL)/streptomycin (100 μg/mL). Cells were maintained under standard conditions at 37 °C in a humidified atmosphere containing 5% CO₂. The cells were passaged when they reached approximately 80–90% confluency. For passaging, cells were rinsed with sterile phosphate-buffered saline (PBS) and detached using 0.25% trypsin–EDTA solution at 37 °C for 2–3 min. For all experiments, freeze-dried extracts were reconstituted in DI water and used to treat cells.

### 2.5. Cytotoxicity Assay

The cellular viability of HCT 116-CCL-247 was evaluated using the tetrazolium MTT (3-(4,5-dimethylthiazol-2-yl)-2,5-diphenyltetrazolium bromide) reduction assay [23]. In each well of a 96-well plate, the cells were seeded at a density of 1 × 10 × 4 cells and allowed to adhere overnight in a growth medium. After a 16 h incubation period, the media were replaced, and the cells were exposed to DI water (as vehicle control) and various concentrations (25, 50, 75, and 100 μg/mL) of aqueous MA-OHE for 6, 12, and 24 h. After the specified incubation times, 10 μL of MTT solution (5 mg/mL in PBS) was added to each well, and the plate was incubated at 37 °C for 2 h. Subsequently, after aspirating to the media, 200 μL of DMSO was added to each well to dissolve the violet formazan crystals. Absorbance was measured at 590 nm using a PerkinElmer Victor X5 microplate spectrophotometer (Victor 5, PerkinElmer, Shelton, WA, USA). This methodology allowed for the assessment of cellular viability under different treatment conditions.

### 2.6. HPLC for Flavonoid Identification

Chromatographic separation was conducted using the HPLC Waters Alliance 2695 system consisting of a quaternary pump, degasser, automatic sample injector, and a C18 column (15 cm × 4 mm, 5 µm, Waters), with the column temperature set at 30 °C and a flow rate of 1 mL/min and a Waters 2487 dual spectrophotometric detector. The mobile phase, consisting of solvent A (DI water + 0.5% phosphoric acid) and solvent B (MeOH 65% + DI water 34.5 + 0.5% phosphoric acid), was prepared and degassed before use. The wavelength was set at 240 nm, and 10 µL of the sample was injected into the chromatographic system. Flavonoids and standards were eluted using the following gradient elution conditions: 90–65% phase A for 0–5 min; 85–70% linear gradient phase A for 5–10 min; 70–40% phase A for 10–11 min; 40–0% linear gradient phase A for 11–12 min; 0–100% reverse linear gradient of phase A for 12–13 min; 100–0% linear gradient phase A for 13–14 min; 50–0% linear gradient phase A for 15–17 min; 50–0% linear gradient phase A for 18–25 min; and 0–20% linear gradient phase A for 25–30 min [24].

### 2.7. HPLC for Fractionation of Flavonoids

Extracts of MA-OHE obtained via water–methanol extraction (WME) and water extraction (WE) were injected into the Waters 600 HPLC System to perform fractionation. Peaks obtained during gradient elution were collected manually using the Waters 600 HPLC System. Each fraction was stored at 4 °C until further analysis. The collected fractions were re-injected into the Waters Alliance 2695 HPLC System to validate the fractionation. Standard compounds corresponding to major peaks were co-injected to confirm the accuracy of the separation process. Chromatograms obtained from HPLC were compared to ensure the reliability of the fractionation process. The peaks were analyzed for retention time consistency and matched with the standards.

### 2.8. Statistical Analysis

One-way ANOVA and Duncan’s multiple range test were used in SPSS Statistics 27.0 for statistical assessment of the data. The results are displayed as mean ± standard deviation, with a 95% confidence level (*p* < 0.05).

## 3. Results and Discussion

### 3.1. HPLC Analysis and Radical Scavenging Activity

**Figure 2:**
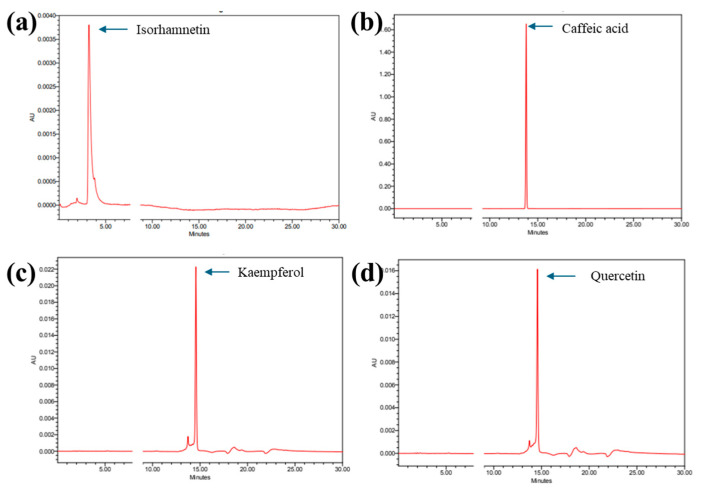
HPLC retention times of standards: (**a**) isorhamnetin; (**b**) caffeic acid; (**c**) kaempferol; (**d**) quercetin.

**Figure 3:**
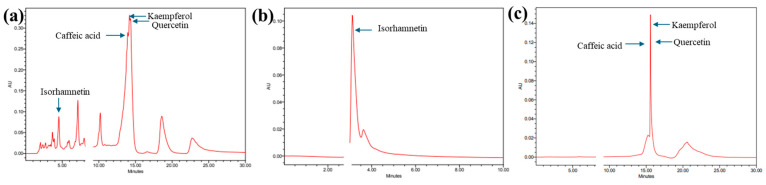
HPLC peaks of (**a**) MA-OHE, (**b**) isorhamnetin after fractionation, and (**c**) caffeic acid, kaempferol, and quercetin (combined) after fractionation.

Chromatographic separation was conducted to elute the flavonoids and standards. The peaks at 240 nm are shown in Figure 2. Standard retention times were observed at 3.221, 13.794, 14.564, and 14.575 for isorhamnetin (Figure 2a), caffeic acid (Figure 2b), kaempferol (Figure 2c), and quercetin (Figure 2d), respectively. The HPLC retention times of the MA-OHE peaks were compared with that of standards, and the presence of the flavonoids was confirmed (Figure 3a). Furthermore, component fractionation and validation of MA-OHE extracts were performed to separate the flavonoids into individual components. Figure 3b shows the fractionated isorhamnetin peak. Since the peaks of caffeic acid, kaempferol, and quercetin have similar retention times, all three flavonoids were fractionated together, as shown in Figure 3c. The HPLC chromatograms demonstrate the successful fractionation of individual flavonoids from the MA-OHE compound.

**Figure 4:**
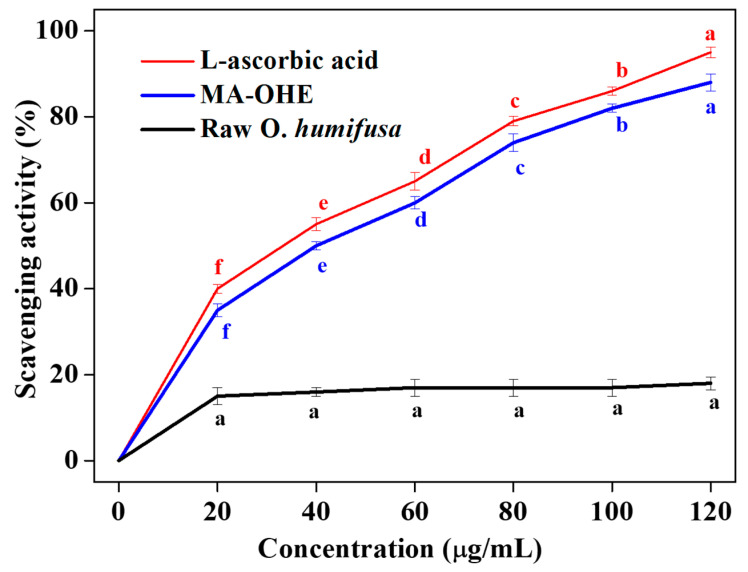
Antioxidant activity of MA-OHE with the standard deviation of the mean derived from three independent experiments (a, b, c, d, e, and f indicate significant differences, *p* < 0.05).

The radical scavenging activity of the MA-OHE was assessed using the DPPH assay. It was confirmed that the aqueous MA-OHE exhibited antioxidant activity in a concentration-dependent manner (Figure 4). The error bars represent the standard deviation of the mean derived from three independent experiments, highlighting the variability in the data. Statistical analyses were conducted to evaluate the differences between the groups, with significant differences (*p* < 0.05) denoted by the letters a, b, c, d, e, and f. These annotations indicate pairwise comparisons at each time point, ensuring clarity in interpreting the observed trends and statistical differences among the three groups. The EC_50_ (IC_50_) values represent the concentration of the plant extract required to achieve 50% inhibition. As the concentration of the extract increased, the inhibition percentages rose, but the EC_50_ values remained consistent, reflecting the concentration at which half of the maximum inhibitory effect was observed. DPPH inhibitory assays were carried out in triplicates, and the values were expressed as mean ± standard deviation. We calculated the IC_50_ values, and the result showed that the MA-OHE extract achieved the IC_50_ at 40 ± 0.92 μg/mL concentration, with an R^2^ value of 0.9221. The raw *O. humifusa* did not reach the IC_50_ value even at a concentration of 120 µg/mL, whereas ascorbic acid had an IC_50_ value of 34 ± 0.12 µg/mL. Notably, the microwave-assisted extract exhibited superior antioxidant activity compared to extracts prepared using conventional methods. This superiority is likely attributable to the reduction in viscosity induced by the high-energy heating of molecules through microwave irradiation, which employs a dual mechanism involving ionic conduction and dipole rotation. The antioxidant activity of MA-OHE is presumed to be linked to its total phenol and flavonoid contents. These components are expected to contribute significantly to their ability to neutralize reactive oxygen species and free radicals. Consequently, these antioxidants play a preventive role by scavenging reactive oxygen species and free radicals.

### 3.2. Effect of MA-OHE on Cytotoxicity and Viability

**Figure 5:**
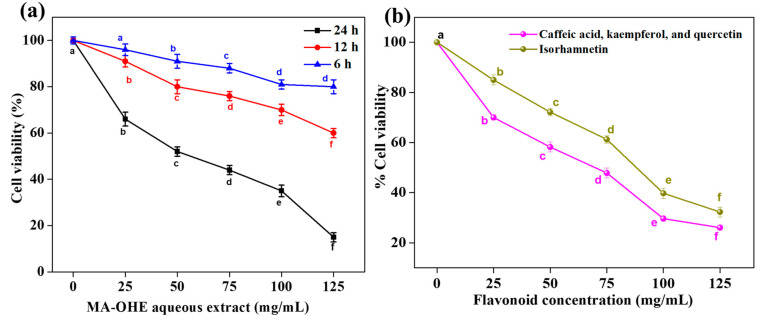
(**a**) Inhibition of HCT 116-CCL-247 cancer cell proliferation by the MA-OHE at different time points. (**b**) Percentage of HCT 116-CCL-247 cell viability after incubation with fractionated flavonoids for 24 h. Both figures show a standard deviation of the mean derived from three independent experiments (a, b, c, d, e, and f indicate significant differences, *p* < 0.05).

To assess the anti-cancer activity of the MA-OHE, different concentrations (25, 50, 75, 100, and 125 mg/mL) of the extract were tested on both the HCT 116-CCL-247 cancer cell line and the healthy HDFa cell line for 6, 12, and 24 h using the MTT assay. Notably, all five extract concentrations demonstrated noticeable cytotoxic effects on the HCT 116-CCL-247 cell line after a 24 h incubation period (Figure 5a). The number of viable cells decreased, and the number of dead cells increased with an increase in both the duration and concentration of the aqueous extract. Cell viability decreased in a time-dependent manner in cells treated with the aqueous extract. MA-OHE extract achieved the IC_50_ at 52 ± 2.02 mg/mL concentration for the 24 h study. The treatment progressively diminished the population of viable cells during the 24 h incubation period in the HCT 116-CCL-247 cell line. The 24-h period of MA-OHE with a concentration of 125 mg/mL reduced the viable cells to 15 ± 2%. Furthermore, we fractionated the flavonoids into individual components and studied the cytotoxic effects. The isorhamnetin with a concentration of 125 mg/mL showed 32% cell viability after 24 h, whereas caffeic acid, kaempferol, and quercetin (combined), after fractionation, showed 26% cell viability over the same time period Figure 5b. Figure 5a,b show a significant decrease in cell viability with concentration (*p* < 0.05), and the error bars represent the standard deviations of three independent experiments.

**Figure 6:**
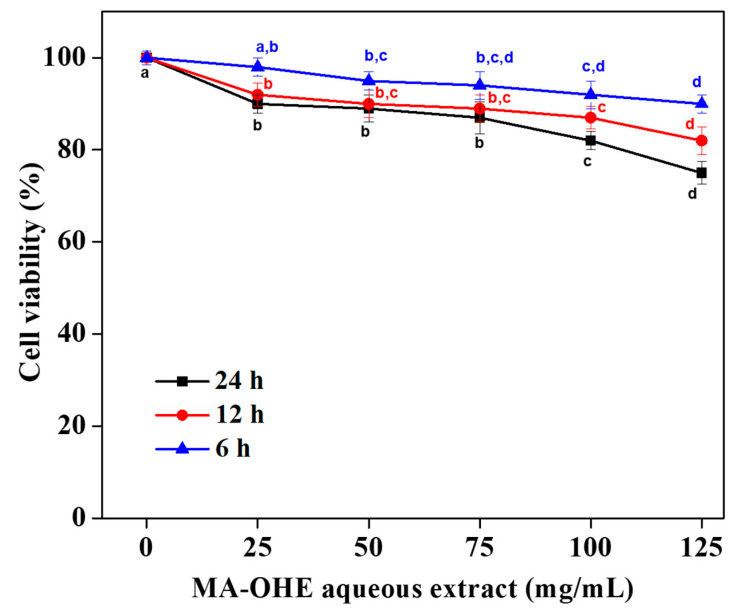
Inhibition of HDFa cell proliferation by the MA-OHE at different time points, with the standard deviation of the mean derived from three independent experiments (a, b, c, and d indicate significant differences, *p* < 0.05).

Conversely, HDFa cells demonstrated a higher survival rate even after exposure to the aqueous extract for 24 h, indicating minimal or negligible cell death in healthy cell lines (Figure 6). In summary, heightened toxicity and cell death were observed in the HCT 116-CCL-247 cell line, particularly at a concentration of 125 mg/mL; in contrast, minimal or reduced cell death, around 75 ± 2.5%, was observed in the HDFa cells at the same concentration. The inhibition of HDFa cell proliferation was not too drastic and is represented by differences between the groups (with *p* < 0.05 representing significant differences). Here, we chose to perform a one-way ANOVA followed by Duncan’s multiple range test for statistical analysis of the data to assess the significance of differences between the experimental groups. One-way ANOVA is appropriate for comparing the means of more than two groups, which is essential for our study given the multiple conditions tested. Duncan’s multiple range test was selected as a post-hoc test to identify specific group differences following the ANOVA, providing a detailed comparison when the overall ANOVA indicated significant differences.

Colon cancer, the third leading cause of mortality in Korea, has prompted researchers to explore its contributing factors in depth. The imperative nature of this inquiry arises from alarming statistics that underscore the need for a comprehensive understanding of the disease [25]. Central to this understanding is the recognition that dietary choices significantly influence the development of colon cancer. A diet rich in fat and red meat, coupled with a deficiency in dietary fiber, fruits, and vegetables, has emerged as a prominent risk factor for colon cancer. The juxtaposition of dietary habits against a backdrop of rising colon cancer rates has prompted a paradigm shift in contemporary research [25,26,27]. Recognizing the limitations and adverse reactions associated with synthetic compounds, particularly in disease treatment, has led to a compelling redirection of focus toward natural products, with particular emphasis on plant extracts. The increasing interest in natural compounds with therapeutic potential extracted from various plants has shown a distinctive upward trend in the last decade. This heightened enthusiasm is driven by awareness regarding the limitations inherent to synthetic compounds and their potential adverse effects, especially in the treatment of complex diseases such as cancer. The increase in the global population has accentuated the demand for alternative treatment sources, prompting extensive exploration of the medicinal properties of plant extracts [3,5,6]. This exploration represents not only scientific curiosity but also a strategic response to healthcare challenges, with a deliberate effort to minimize the potential drawbacks associated with synthetic pharmaceuticals.

Among the myriad plant extracts under investigation, *O. humifusa*, a particular variety of prickly pear cactus, has emerged as a promising candidate. Revered for harboring biologically active compounds with potential therapeutic applications, *O. humifusa* holds promise for treating conditions such as diabetes mellitus, arteriosclerosis, and hyperglycemia [28]. This study aimed to investigate the effects of microwave-assisted *O. humifusa* aqueous extract on the proliferation and cell death of human colon carcinoma HCT 116-CCL-247 cells. Notably, *O. humifusa* has a diverse composition, characterized by its richness in carbohydrates, essential minerals (g, K, and Ca), flavonoids, and total phenolics. HPLC was employed to identify specific flavonoids, revealing a distinct profile that included isorhamnetin, caffeic acid, kaempferol, and quercetin. Based on this knowledge, we attempted to assess the impact of microwave-assisted *O. humifusa* aqueous extracts on HCT 116-CCL-247 cells. The experiments involved treating these colon cancer cells with varying concentrations of the extract (ranging from 25 to 125 mg/mL) over a 24 h period. The results revealed remarkable dose-dependent cell growth inhibition. What made this discovery even more compelling was its specificity. This inhibitory effect was conspicuously absent in HDFa cells, underscoring the potential selectivity of *O. humifusa* in targeting colon cancer cells. In this study, we focused on oxidative stress, a well-explored phenomenon recognized for its deleterious effects on cellular components. This study revealed the antioxidant potential of plant-derived phenolic compounds, particularly flavonoids, as a promising avenue in the context of anti-cancer activity. These compounds exhibit a remarkable ability to counteract oxidative stress, acting as a protective shield against damage inflicted upon cellular components that typically culminates in uncontrolled cell growth. The distinctive aspect of this revelation lies in the specificity of the observed growth inhibition confined to colon cancer cells (HCT 116-CCL-247) while leaving HDFa cells untouched. This specificity has profound implications and suggests the potential use of these flavonoids as targeted agents against colon cancer. The development of agents capable of selectively impeding cancerous cells while sparing normal healthy cells is a critical step in the pursuit of effective and precise cancer therapies.

In the experimental section, we applied the HPLC method to analyze and identify the bioactive compounds present in the *O. humifusa* extract. The HPLC analysis identified key flavonoids, which were identified and fractionated based on their retention times and peak areas. While these findings provided valuable insights into the composition, further optimization of the HPLC method is needed to address peak overlap issues and compound quantification. The ability of *Opuntia humifusa* extracts to inhibit colon cancer cell proliferation is consistent with previous studies that have demonstrated the anticancer properties of flavonoids [29]. The extract demonstrated strong antioxidant activity in the DPPH assay, suggesting that it contains bioactive compounds capable of modulating oxidative stress. In cancer cells, this modulation can disrupt the redox balance, leading to the generation of reactive oxygen species (ROS) at levels that induce cellular damage and impair mitochondrial function. The MTT assay results suggest mitochondrial activity is significantly inhibited in cancer cells treated with the extract. The enhanced bioactivity of the processed extract compared to the raw *O. humifusa* implies that the processing step may increase the bioavailability or potency of specific active compounds, which contribute to oxidative stress modulation and cell death pathways in cancer cells [30,31].

## 4. Conclusions

This study highlights the potential of *O. humifusa* aqueous extract (MA-OHE) as a promising candidate for colon cancer treatment. The extract demonstrated a dose-dependent inhibition of HCT 116 colon cancer cell proliferation, with significant cytotoxicity observed at an IC_50_ concentration of 52 ± 2.02 mg/mL after 24 h. Importantly, the extract exhibited minimal cytotoxicity in healthy HDFa cells, indicating selective anti-cancer properties. Additionally, the enhanced antioxidant activity of the processed extract, as demonstrated by its IC_50_ value of 40 ± 0.92 µg/mL in DPPH analysis, underscores the role of processing in amplifying its bioactivity compared to raw *O. humifusa*. While the study provides compelling evidence of the selective anti-cancer potential of MA-OHE, further investigations are required to elucidate the mechanisms of cell death and validate these findings through apoptosis-specific assays. Moreover, the isolation and cytotoxicity testing of individual flavonoid components presents new opportunities to identify active compounds with therapeutic potential. These findings pave the way for leveraging natural products as a foundation for innovative and targeted cancer treatments, bridging the gap between traditional knowledge and modern therapeutic strategies.

## Data Availability

The original data presented in this work are included in the article; further inquiries can be directed to the corresponding author.
